# Effects of dietary phytosterols or phytosterol esters supplementation on growth performance, biochemical blood indices and intestinal flora of C_57_BL/_6_ mice

**DOI:** 10.1371/journal.pone.0297788

**Published:** 2024-05-14

**Authors:** Wenxin Ye, Wenzi Wu, Lai Jiang, Chunchun Yuan, Yubo Huang, Zhuo Chen, Qixin Huang, Lichun Qian

**Affiliations:** 1 Hainan Institute of Zhejiang University, Sanya, China; 2 Key Laboratory of Animal Nutrition and Feed Science in East China, Ministry of Agriculture, College of Animal Sciences, Zhejiang University, Hangzhou, China; University of Brescia: Universita degli Studi di Brescia, ITALY

## Abstract

This study was conducted to evaluate the effects of phytosterols (PS) and phytosterol esters (PSE) on C_57_BL/_6_ mice. Three groups of 34 six-week-old C_57_BL/_6_ mice of specific pathogen free (SPF) grade, with an average initial body weight (IBW) of 17.7g, were fed for 24 days either natural-ingredient diets without supplements or diets supplemented with 89 mg/kg PS or diets supplemented with 400 mg/kg PSE. Growth performance, blood biochemistry, liver and colon morphology as well as intestinal flora status were evaluated. Both PS and PSE exhibited growth promotion and feed digestibility in mice. In blood biochemistry, the addition of both PS and PSE to the diet resulted in a significant decrease in Total Cholesterol (TC) and Triglyceride (TG) levels and an increase in Superoxide Dismutase (SOD) activity. No significant changes in liver and intestinal morphology were observed. Both increased the level of Akkermansia in the intestinal tract of mice. There was no significant difference between the effects of PS and PSE. It was concluded that dietary PS and PSE supplementation could improve growth performance, immune performance and gut microbiome structure in mice, providing insights into its application as a potential feed additive in animals production.

## 1 Introduction

Phytosterol is a natural component of plant oil, which is a functional component existing in the free state or combined with sugar and other states. They are usually extracted from deodorized distillates produced during the refining of vegetable oils or from tall oil, which is a by-product of the paper pulp industry [1,2]. It mainly includes β-sitosterol, soy sterol, rape oil sterol and other natural physiologically active substances with a cyclopentane allhydrophil as the backbone [3,4]. Because it can significantly reduce the cholesterol level of the body and has a unique antioxidant, growth promotion, immune regulation, hormone-like, and other important physiological functions, PS are called "the key to life" by scientists. People have been studying PS for hundreds of years, and its physiological functions such as lowering cholesterol and preventing cardiovascular diseases have been proved in the early stage [5,6].

PS have preventive and therapeutic effects on various chronic diseases, such as Cardiovascular Diseases (CVD), hepatoprotection, diabetes and cancer [7–11]. The intake of PS-rich foods or supplements are considered a good dietary treatment to reduce the risk of CVD [12]. It has even been shown that PS are associated with cognitive function and are expected to be used in the prevention and treatment of Alzheimer’s disease [13–16].

However, the unique chemical structure of PS determines their high melting point (>130°C) and low solubility in water and edible oils, limiting their applications in the food, medical, cosmetic, and other industries. To overcome this problem, the most common and effective solution is the synthesis of the corresponding fatty acid esters of PS. The esterified form exhibits higher solubility in oil, a lower melting point, while retaining all the excellent properties of the original PS [17–20]. On the one hand, fatty acid esters of PS can be easily incorporated into various diets and fatty foods. On the other hand, numerous studies in recent years have confirmed that PSE have many functions similar to those of PS [21]. Esterified PS can effectively lower blood TC and Low-Density Lipoprotein Cholesterol (LDL-C) levels in a similar manner to free PS [19]. PSE can be synthesized by the reaction of PS with fatty acids or their derivatives, mainly by chemical synthesis and enzyme-catalyzed synthesis. It was shown that PSE effective in stopping progression of nonalcoholic fatty liver disease (NAFLD) [22]. PSE promote muscle development in hen laying chicks hatching by regulating Bile Acid (BA) deposition [23].

In the available literature, there is no information on the effects of PS and PSE on gastrointestinal flora. Microbial activity in the gut is important because gut bacteria are involved in the regulation of many metabolic pathways, initiating host-microbe interactions that connect the gut, liver, muscle and brain [24,25]. Changing ration composition can lead to changes in substrate influx in the coliforms. The addition of PS and PSE to the diet may affect the microbial composition and activity in the gut, which in turn may affect the physiology of the animal. Therefore, this study aims to evaluate the effects of adding PS and PSE to the diet on the growth performance, blood physiology and biochemistry, as well as the intestinal microbial composition in C_56_BL/_6_ mice.

## 2 Materials & methods

All procedures of animal experiments were carried out based on protocols approved by the Animal Care Advisory Committee of Zhejiang University, Hangzhou, China (No. ZJU20210097).

**Animal Welfare Statement**:

The authors confirm that the ethical policies of the journal, as noted on the journal’s author guidelines page, have been adhered to and the appropriate ethical review committee approval has been received. The authors confirm that they have followed EU standards for the protection of animals used for scientific purposes [and feed legislation, if appropriate].

### 2.1 Animals and nutrition

The experiment was performed on 34 six-week-old male C_57_BL/_6_ mice of SPF grade divided into 3 groups with an average initial body weight of 17.713g. Housed for 24 days in individually ventilated cages with *ad libitum* access to food and water, all experiments were conducted during photoperiod. Mice in control (CK) group were fed with a commercial, cereal-based diet for laboratory animals (Jiangsu Synergy Medicine Bioengineering Co., Ltd, China), the mice in the first experimental (T1) group were fed with a commercial, cereal-based diet for laboratory animals adding 89 mg/kg PS (Zhejiang Delekang Food Co., Ltd., Zhejiang, China) and the mice in the second experimental (T2) group were fed with a commercial, cereal-based diet for laboratory animals adding 400 mg/kg PSE (CN115286677A, China). Previous studies have found that the supplementation of PS at a dosage of 80mg/kg to the diet had the most significant effect in increasing the growth rate of broilers [26], combined with the amount of synthesized PSE in the same dosage, we set our additions at 89 mg/kg of PS and 400 mg/kg of PSE. The composition and nutrient level of the basal diet of three groups are shown in Table 1. The experimental period was 24 days. The content of PS and PSE in the feed is a comprehensive reference to previous experiments and articles, and the amount of PSE added is converted to the equivalent content of PS [27].

**Table 1:** Composition and nutrient level of the basal diet (air-dry basis).

Composition(g/kg)	CK	T1	T2
Water	94.1	94.1	94.1
Crude protein	209	209	209
Crude fat	49	49	49
Crude fiber	21	21	21
Crude ash	54	54	54
Calcium	12	12	12
Total phosphorus	7.7	7.7	7.7
Calcium: Total Phosphorus	1.56:1	1.56:1	1.56:1
Lysine	16.9	16.9	16.9
Methionine plus Cystine	16.9	16.9	16.9
PS	/	0.089	/
Phtosterol esters	/	/	0.4

### 2.2 Sample collection

At the end of the experiment (24 days old), the finishing mice were fasted for 6-hour daytime and 16-hour overnight [28] and at which time, blood samples were collected at baseline into a vacuum tube. Mice were anesthetized by intraperitoneal injection of 80 mg/kg sodium pentobarbital and were euthanized by intraperitoneal injection of 200 mg/kg sodium pentobarbital under deep anesthesia. The blood samples were centrifuged at 3500 x g for 15 minutes at 4°C, and then serum was separated and stored at -20°C for further analysis. Then the mice in three groups were humanely euthanized according to the recommendations of the animal welfare literature. The sample numbers of the liver and colon of the finishing mice were placed in the sample bag, and the contents of the colon were loaded into a 1.5 ml eppendorf tube. The sample bag and the eppendorf tube were wrapped with a suitable tin foil paper and placed in a liquid nitrogen tank for temporary storage [29]. Then transferred to—80°C refrigerator for further testing.

### 2.3 Growth performance indicators

The mice and food were weighed on the 1^st^, 11^th^, and 24^th^ days. Daily weight gain and daily feed intake were calculated.

### 2.4 Histology

Colon tissue and liver were made into standard wax blocks for sectioning after washing, dehydration and transparency, and wax embedding. The slice thickness was 3 μm. The cut tissue pieces were placed on glass slides, dried in a 37°C incubator, and then placed in a glass slide box for later use. Intestinal Hematoxylin-eosin (HE) staining slices were at 100x magnification, and 3 fields of view were randomly selected for each slice for image acquisition, with 10 slices in each group. Intestinal villus height and crypt length were calculated using Image Pro Plus 6.0. Using 200 x magnification, 3 fields of view were randomly selected for each liver HE stained slices for image acquisition, with 10 slices in each group. Observe whether the shape of liver cells was complete, if the nucleus is enlarged, and whether the shape of liver lobules appeared intact, etc.

### 2.5 Serum biochemical indicators

The level of Albumin (ALB), Total Protein (TP), TC, TG, Aspartate Aminotransferase (AST), Alanine Aminotransferase (ALT) and SOD of the mice were measured using assay kits with Ultraviolet–visible spectroscopy (UV-VIS) Spectrophotometer according to the manufacturer’s instructions (Nanjing Jiancheng Institute of Bioengineering, China). The inflammatory markers of the mice (MEIMIAN, Jiangsu, China) including Immunoglobulin A (IgA), Interleukin-1β (IL-1β), Interleukin-6 (IL-6), Interleukin-10 (IL-10), Interleukin-22 (IL-22), Tumor Necrosis Factor-α (TNF-α). And the neutrophil levels including Chemokine (C-X-C motif) Ligand 1 (CXCL-1) and Chemokine (C-X-C motif) Ligand 2 (CXCL-2) were measured with assay kits according to the manufacturer’s instructions (Nanjing Jiancheng Institute of Bioengineering, China) with UV-VIS Spectrophotometer (UV1100, MAPADA, Shanghai, China According to the manufacturer’s instructions.

### 2.6 Microbial 16s RNA gene sequencing library construction and sequencing

The 16s RNA sequencing and bioinformatics analysis were performed by Majorbio (Shanghai, China). The resulting sequences were clustered into operational taxonomic units (OTUs) using Uparse drive5 (version 7.0.1090) at 97% sequence similarity. Extract non-repetitive sequences from the optimized sequences to facilitate the reduction of redundant computation in the middle process of analysis, remove the single sequences without repetition, perform OTU clustering of non-repetitive sequences (excluding single sequences) according to 97% similarity, remove chimera in the clustering process to obtain representative sequences of OTU, map all optimized sequences to OTU representative sequences, select sequences with similarity of 97% or more to the representative sequences, and generate OTU tables. Alpha diversity indices (including the Sobs index, Chao richness estimator, Shannon diversity index, and Simpson index) between the mice of three groups were calculated to evaluate microbial species evenness (Mojorbio, Shanghai, China). Beta diversity was evaluated by principle coordinate analysis (including PCoA index and non-metric multidimensional scaling index) based on the unweighted UniFrac distance [1] (Mojorbio, Shanghai, China). Use Wilcox rank-sum test analysis to analyze which bacteria differ in the intestinal flora of different groups of mice at the phylum level and genus level, respectively. The raw sequencing reads were deposited into the NCBI Sequence Read Archive (SRA) database (Accession Number: PRJNA905562).

#### 2.6.1 Environmental factor correlation analysis: Correlation heatmap plots

The indicators ALB, TP quantitative, IgA, SOD, CXCL1, CXCL2, AST and ALT were analyzed for environmental factor correlations with the intestinal flora of three groups of mice using Spearman correlation, a non-parametric version of the Pearson’s correlations. Correlations between microbial taxa and environmental variables were assessed by correlation heatmap plots visualizing the relationship between different species in the samples and environmental variables through correlation values.

### 2.7 Statistical analysis

All data except for the 16s RNA results were analyzed by one-way analysis of variance (ANOVA) using SPSS statistical software (Ver. 20.0 for windows, SPSS, Inc., Chicago, IL, USA). Differences among treatments were examined using the Tukey-Kramer’s multiple range tests, which were considered significant when the p-value was less than 0.05. Results are presented as means alongside their pooled standard errors of means (SEM).

## 3 Results

### 3.1 Change in average feed intake and body weight

The changes in body weight and mean food intake of mice in each group before and after the experiment are shown in Table 2. The results showed that the average daily weight gain and food intake of mice in both the PS and PSE treated groups were significantly higher compared to those in the control group. This indicates that PS and PSE have the effect of improving the growth performance of mice to a certain extent.

**Table 2:** Effects of PS and PSE on growth performance of mice.

Items	Treatment			SEM	*P-Value*
	CK	T1	T2		
IBM (g)	17.885 ± 0.144	17.618 ± 0.097	17.636 ± 0.046	0.676	0.208
FBW (g)	20.845^b^ ± 0.299	21.640^a^ ± 0.271	22.033^a^ ± 0.046	0.211	0.030*
ADG (g/d)	0.118^b^ ± 0.018	0.161^a^ ± 0.010	0.176^a^ ± 0.004	0.105	0.034*
ADFI (g/d)	2.610^b^ ± 0.173	3.096^a^ ± 0.070	3.073^a^ ± 0.013	0.096	0.032*
FCR	24.935^a^ ± 4.317	15.851^b^ ± 0.664	14.304^b^ ± 0.349	2.085	0.050*

^a-b^ Means with different superscripts in the same row indicate significantly difference (*p* < 0.05). IBM: Initial body weight; FBW: Final body weight; ADG: Average daily gain; ADFI: Average daily food intake; FCR: Food conversion ratio. CK: Basic diet; T1: Basic diet + 89mg / kg PS; T2: Basic diet + 400mg / kg PSE; SEM: Standard error means.

### 3.2 Effects of PS and PSE on the liver of mice

The liver TC and TG contents, as well as AST and ALT vitality of mice in each group are presented in Table 3. The experimental results showed that the liver TC and TG contents of mice in the PS and PSE groups were significantly higher than those in the control group. There was no significant difference in the activity of AST and ALT, indicating that the plant and sterol esters did not have a significant effect on the liver function of mice. The experimental results showed that the albumin content, SOD vitality of mice in the T1 and T2 groups have increased significantly, among which the effect of improving ALB content of PS was better than those of PSE, indicating that both can effectively improve the immunity of the mice organism, among which the effect of PS is better. And the effect of the increase of SOD vitality of PSE was better than that of PS. both could improve the antioxidant ability of mouse organism and had good effect of lipid peroxidation, and the effect of PSE was greater than PS.

**Table 3:** Effect of PS and PSE on biochemical index in mice.

Items	Treatment			SEM	*P-Value*
	CK	T1	T2		
TC (mmol/L)	0.023^c^ ± 0.002	0.042^a^ ± 0.002	0.032^b^ ± 0.003	0.002	<0.001**
TG (mmol/L)	0.636^b^ ± 0.048	1.501^a^ ± 0.094	1.105^a^ ± 0.073	0.087	<0.001**
AST (U/gprot)	102.475 ± 15.634	89.734 ± 12.412	113.694 ± 29.551	12.213	0.728
ALT (U/gprot)	32.140 ± 2.017	31.378 ± 1.754	33.609 ± 2.390	1.185	0.731
ALB (%)	11.788^b^ ± 0.369	14.367^a^ ± 0.552	13.464^a^ ± 0.554	0.370	0.048
TP (%)	34.936 ± 1.974	28.778 ± 2.574	31.040 ± 1.995	1.438	0.377
SOD (U/ml)	67.947^c^ ± 1.067	74.714^b^ ± 1.434	79.571^a^ ±0.191	1.286	<0.001**

^a-c^ Means with different superscripts in the same row indicate significantly difference (*p* < 0.05). TC: Total cholesterol; TG: Triglyceride; AST: Aspartate aminotransferase; ALT: Alanine aminotransferase; ALB: Albumin; TP: Total protein; T1: Basic diet + 89mg / kg PS; T2: Basic diet + 400mg / kg PSE; SEM: Standard error means.

### 3.3 Effect of PS and PSE on anti-inflammatory properties in mice

The effects of PS and PSE on interleukins in mice are shown in Table 4. The experimental results show that IgA content in the T1 and T2 groups have increased significantly, while CXCL1 and CXCL2 levels were significantly reduced, with a reduction effect: PSE group > PS group. CXCL2 has 90% amino acid homology with the related chemokine CXCL1. This chemokine is secreted by monocytes and macrophages and has a chemotactic effect on polymorphonuclear leukocytes and haematopoietic stem cells. The anti-inflammatory and anti-tumor effects of PS and PSE were demonstrated, and initially the effect of PSE was greater than that of PS.

**Table 4:** Effect of PS and PSE on Interleukin in mice.

Items	Treatment			SEM	*P-Value*
	CK	T1	T2		
IgA (μg/mL)	45.163^b^ ± 2.310	61.184^a^ ± 4.514	59.896^a^^b^ ± 3.540	2.630	0.047*
Mouse IL-1β	47.053 ± 4.435	34.255 ± 5.38	32.828 ± 8.637	3.553	0.172
Mouse IL-22	13.982 ± 1.025	15.885 ± 1.741	16.760 ± 1.223	0.780	0.349
Mouse TNF-α	69.247 ± 2.613	70.213 ± 6.396	63.671 ± 6.194	2.545	0.647
Mouse CXCL1	129.661^a^ ± 8.614	107.063^a^^b^ ± 6.335	98.442^b^ ± 2.764	5.726	0.045*
Mouse CXCL2	328.326^a^ ± 5.513	250.843^b^ ± 22.790	234.215^b^ ± 20.672	14.613	0.002**
Mouse IL-6	108.218 ± 5.967	94.288 ± 4.389	98.106 ± 5.178	3.556	0.222
Mouse IL-10	262.533 ± 25.346	276.046 ± 35.704	310.877 ± 32.956	17.131	0.582

^a-c^ Means with different superscripts in the same row indicate significantly difference (*p* < 0.05). IgA: Immunoglobulin A; SOD: Serum Superoxide Dismutase; CK: Basic diet; Mouse IL-1β: Mouse Interleukin-1β; Mouse IL-22: Mouse Interleukin 22; Mouse TNF-α: Mouse Tumor Necrosis Factor-α; Mouse CXCL1: Chemokine (C-X-C motif) ligand 1; Mouse CXCL2: Chemokine (C-X-C motif) ligand 2; Mouse IL-6: Mouse Interleukin 6; Mouse IL-10: Mouse Interleukin 10; CK: Basic diet; T1: Basic diet + 89mg / kg PS; T2: Basic diet + 400mg / kg PSE; SEM: Standard error means.

### 3.4 Morphological changes in the liver and colon

**Fig 1:**
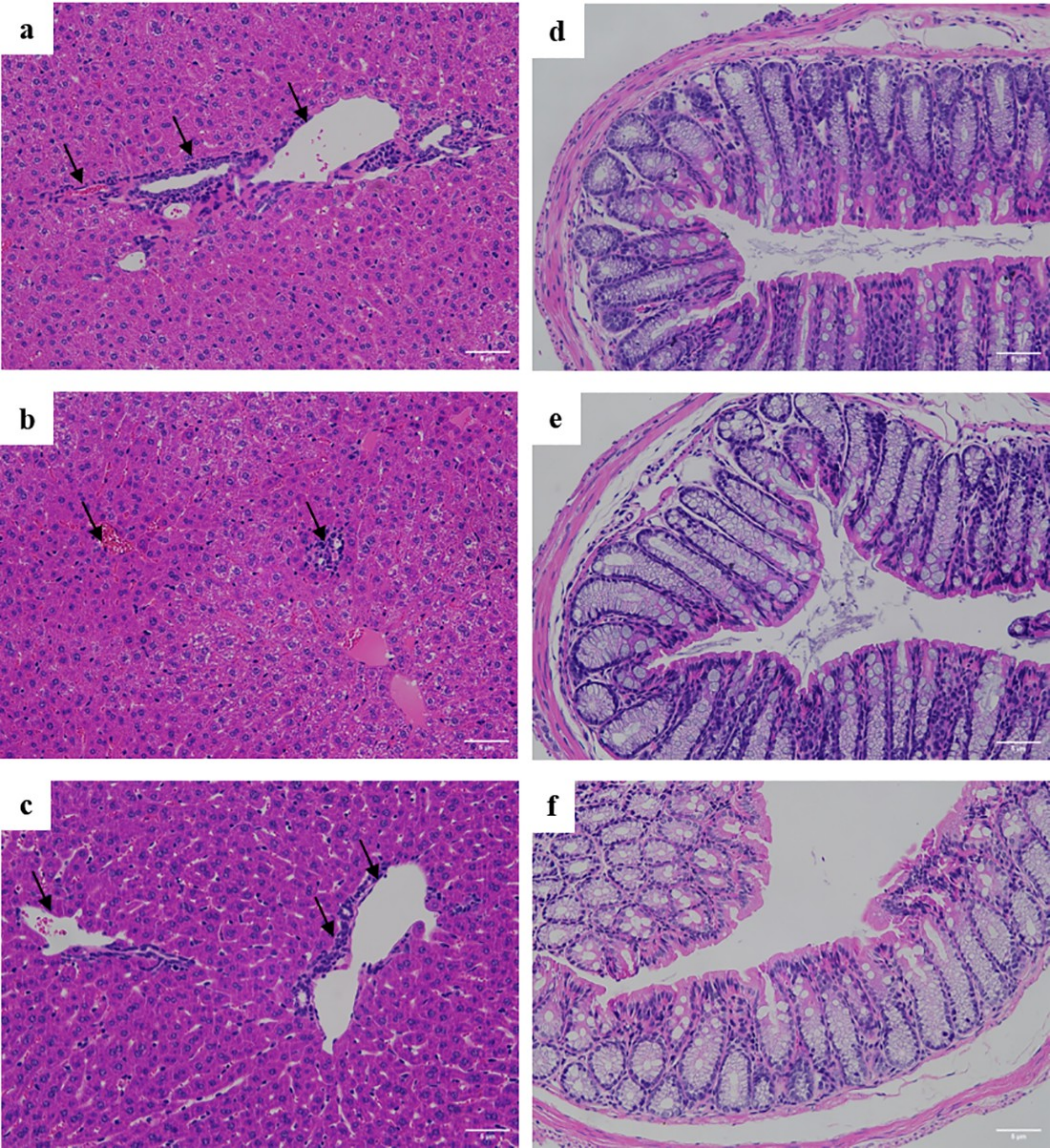
Examples of images of haematoxylin-eosin-stained sections of the liver (a, b, c) and colon (d, e, f) of C_57_BL/_6_ mice fed the control diet (a, d) and diet supplemented with 89mg / kg PS (b, e) and diet supplemented with 400mg / kg PSE (c, f).

There was no effect of diets containing PS or PSE on crypt depth or villi height in the colon, nor on liver cell morphology. Examples of images of haematoxylin-eosin-stained sections of the liver and colon are shown in Fig 1.

### 3.5 Microbial 16s rRNA gene sequencing library construction and sequencing

#### 3.5.1 Changes in intestinal microbial diversity and abundance in mice and similarity of communities between groups

**Fig 2:**
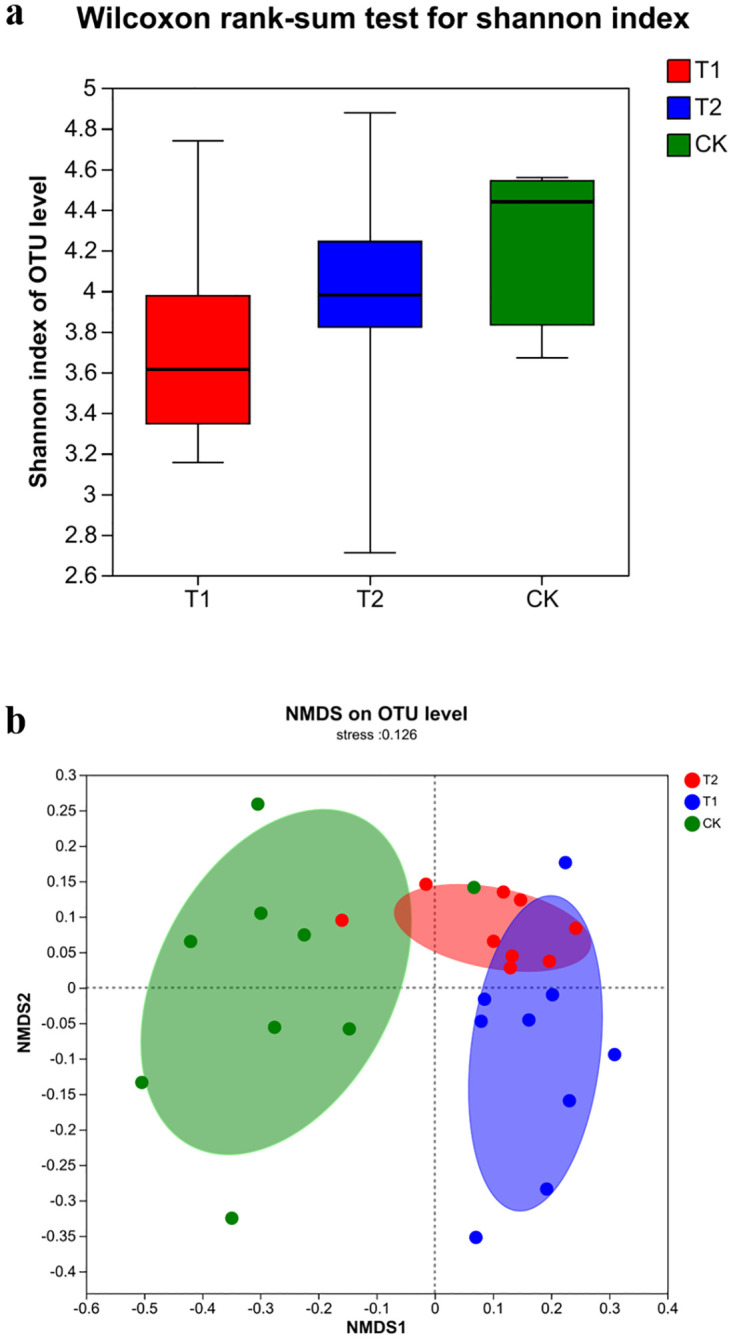
Differences of alpha(a) and NMDS(b) analysis of intestinal flora in mouse of three groups.

Differences between and within groups in gut microbial diversity in mice are demonstrated in Fig 2. From Fig 2a, it can be seen that there are significant differences in the intestinal flora of the three groups of mice, where the diversity of microorganisms is: PSE group > PS group > control group, which shows that PSE and PS can increase the diversity of flora in the intestine of mice. From Fig 2b, it can be seen that the intestinal community species of the three groups of mice were very different.

#### 3.5.2 Wilcox rank sum test results

**Fig 3:**
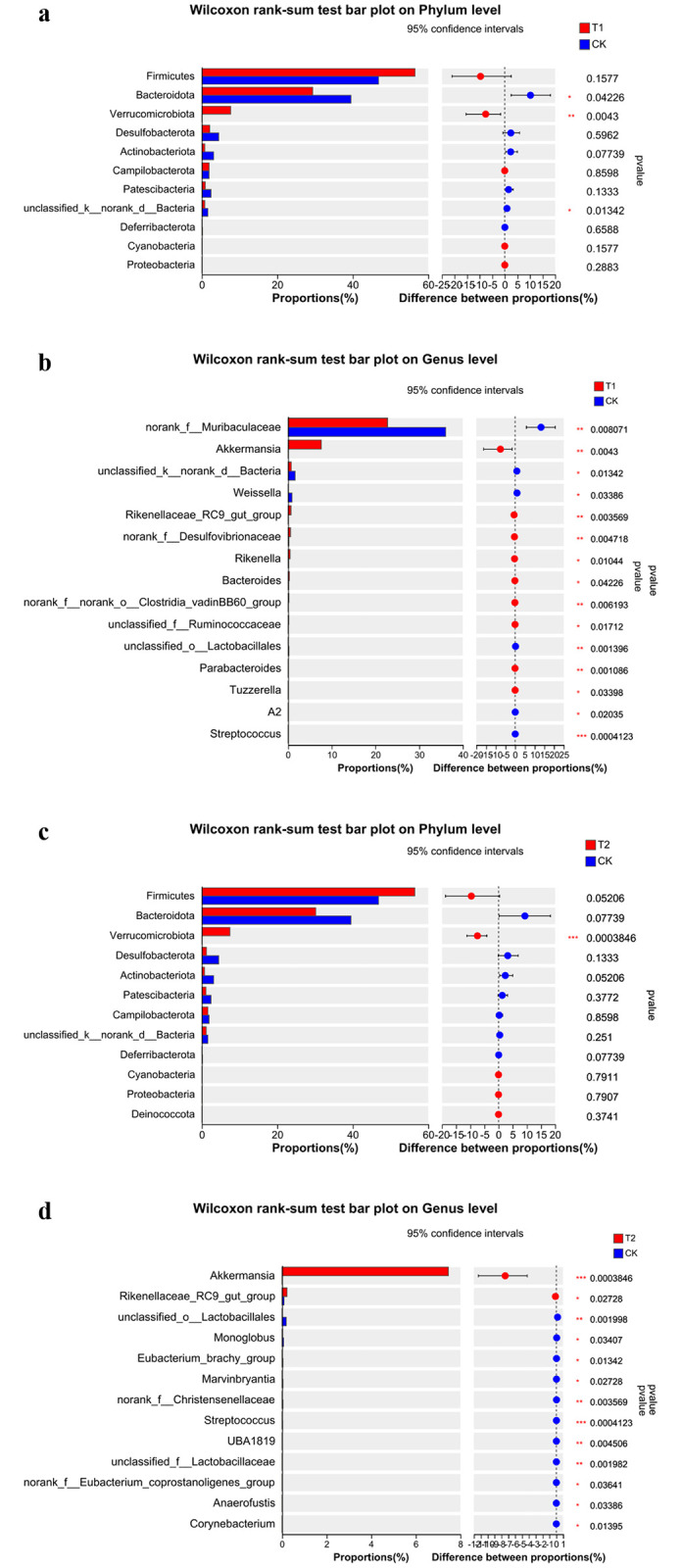
Differences of intestinal flora between mice of T1group and CK group in phylum level (a) and genus level (b). Difference of intestinal flora between mice of T2 group and CK group in phylum level (c) and genus level (d).

Analysis of the differential flora in each colonic content using the Wilcox rank sum test reveals differences between the three groups of mice intestinal microorganisms at the phylum level and the genus level, as shown in Fig 3. Of these, the PSE group showed significantly higher differences in the mice intestinal microorganisms compared to the control group at the phylum level in the phylum Verrucomicrobiota, a phylum of bacteria that has been shortly delineated and includes a few identified species that are found mainly in aquatic and soil environments, or in human feces. There are still relatively few studies on it. While at the genus level, the difference between Akkermansia was significantly higher in mice in the PSE group compared to the control group.

In the PS group compared to the control group, at the phylum level the phylum Bacteroidota and the phylum Verruciformis differed significantly, with the phylum Bacteroidota being significantly lower and the phylum Verruciformis significantly higher. At the genus level Muribaculaceae was significantly lower, and Akkermansia was significantly higher than the control group.

#### 3.5.3 Correlation analysis of some physiological and biochemical indicators with mouse flora at the phylum and genus levels

**Fig 4:**
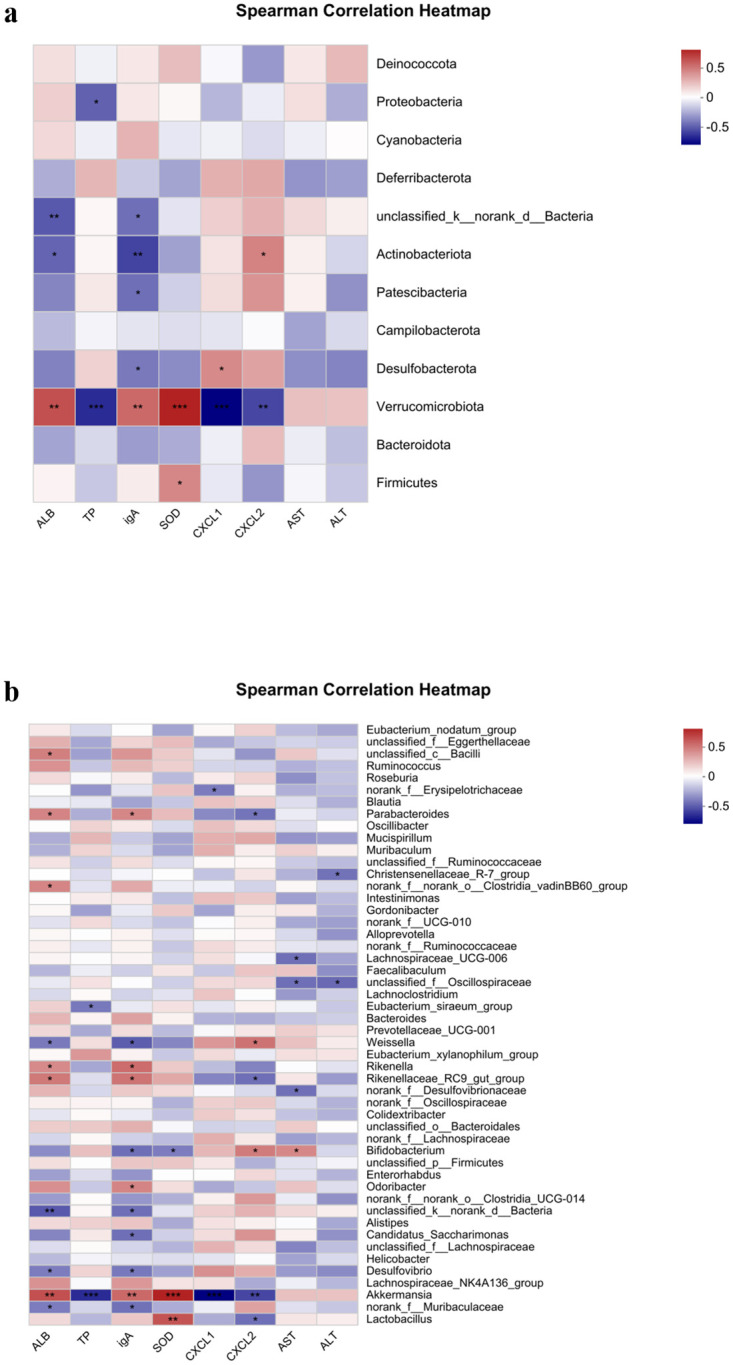
Environmental factor association analysis of three groups of mouse intestinal microorganisms at the phylum (a) level and genus (b) level.

The correlation of some biochemical indicators with the intestinal flora of the three groups of mice was analyzed at the gate level and the genus level respectively (Fig 4). It can be seen that there is some non-linear correlation between the high and low levels of certain indicators and the specific flora.

At the phylum level, the one phylum that can be seen to correlate significantly with the indicators is the phylum Warty Microflora, the number of which correlates significantly and positively with the levels of ALB and IgA and the activity of SOD, and negatively with the levels of TP, CXCL1 and CXCL2 (Fig 4a).

At the genus level, where the activity of SOD was significantly and positively correlated with the number of Akkermansia, the content of ALB and IgA was also significantly and positively correlated with the number of Akkermansia. The number of TP and the content of CXCL1 and CXCL2 were negatively correlated with the number of Akkermansia, while the activity of SOD was also positively correlated with the number of Lactobacillus and the number of Lactobacillus was negatively correlated with the content of CXCL2 (Fig 4b).

## 4 Discussion

PS and PSE have positive effect to growth of mice. Previous studies have only identified the indispensable role of PS for plant growth and development [30], and little research had been done on the role of PS for animal growth and development. From our result of the change in ADFI and FBW we can see that the ADG and ADFI of mice fed with diets adding PS and PSE (Group T1 and Group T2) are significantly higher than the mice in the control group (CK). This proved that PS and PSE can significantly promote the growth and development rate and increase the daily food intake of mice. In addition, the FCR of mice fed diets supplemented with PS and PSE was also significantly lower than that of mice in the control group, suggesting that the addition of PS and PSE to the diets can effectively improve feed utilization in mice. Compared to previous studies, the present study adds an exploration of the multifaceted effects of PSE intake, providing implications and a basis for PSE utilization.

The effects of PS intake on TC and TG in mice is in doubt. According to the results of our three groups of mice in terms of differences in several liver function indicators of TG, TC, AST and ALT, although there was no significant difference in AST and ALT activity of the three groups of mice, which means that PS and PSE have no significant effect on mice in liver circulation and metabolism and hepatocyte damage, but there is a significant difference in TC and TG content of the three groups, and the TC and TG content of mice fed PS and PSE are significantly higher than those of mice in control group. As early as the 1950s, PS were known to lower TC, especially LDL-C, and their cholesterol-lowering effects were mediated by competition for cholesterol ligands. From then on a growing number of studies have also demonstrated that PS are effective in reducing cholesterol in hyperlipidemic patients as well as in hyperlipidemic mice [31–33]. Also, Study finds intake of PS effective in reducing TG levels in humans [34]. Our experimental results, however, were contrary to these findings, showing that both TC and TG were significantly elevated in mice that had ingested PS. The occurrence of this result may be due to the fact that the mice in the two experimental groups grew too fast compared to the control group, indicating that the growth-promoting effects of PS and PSE on mice are mainly in the promotion of fat synthesis. Researches have also found that Rikenellaceae_ RC9_ gut_group is related to high fat diet and could improve the levels of TG and TC in female offspring at weaning [35,36]. In our study, Rikenellaceae_ RC9_ gut_group bacteria were also significantly increased in the intestines of mice receiving PS and PSE. So PS and PSE might promote fat metabolism by increasing the amount of Rikenellaceae_ RC9_ gut_group in the intestine, which improves the growth rate and body weight of mice.

PS and PSE have positive effects in improving anti-inflammatory and antioxidant properties in mice. The chemokines CXCL1 and CXCL2, as inflammatory mediators, are highly expressed in response to inflammation [37–42]. The results of the study showed that CXCL1 and CXCL2 were significantly decreased in mice of both T1 and T2 groups, which showed that the intake of PS and PSE could effectively alleviate the inflammatory symptoms in mice. IgA neutralizes and blocks the activity of viruses, bacteria and protozoa [43], and also interacts with other innate defense factors in mucosal secretions to enhance immune protection [44,45]. The results showed that the IgA content of mice in T1 and T2 groups was significantly elevated compared to mice in the control group, which showed that PS and PSE have the ability to improve the immunity and antioxidant capacity of the body and effectively prevent lipid peroxidation.

PS and PSE improve health in mice by altering intestinal flora. The level of Akkermansia in the intestinal flora was significantly increased in mice fed with PS in their diets. Akkermansia muciniphila, a newborn probiotic, is abundant in the intestinal flora of healthy individuals and has preventive and therapeutic effects on metabolic dysfunction such as obesity and type 2 diabetes [46–49]. Also, by modulating mucus layer thickness, the intestinal commensal bacterium Akkermansia muciniphila showed a positive association with the lean phenotype, improved metabolic response and restoration of intestinal barrier function [50]. Mice in both T1 and T2 groups had a higher level of Akkermansia compared to the mice in control group. Given that the results of environmental factor correlation analysis showed a significant positive correlation between Akkermansia and SOD activity values, we could say that Akkermansia can attenuate oxidative stress and inflammatory responses by enhancing SOD activity, as demonstrated in previous studies [51]. Similarly, the correlation heat map of environmental factor association analysis showed a significant negative correlation between Akkermansia and both inflammatory chemokines CXCL1 and CXCL2, and it is reasonable to speculate that the effect of Akkermansia in reducing inflammation could be achieved by decreasing the chemokines CXCL1 and CXCL2 [52].

Previous researches have found some connections between PS or PSE diet and gut flora. Feng et al. found that a PS diet increased Bacteroidetes levels in Topaz broilers [53]. Lv et al. found that dietary addition of PS improves the growth of rumen bacteria and alters rumen fermentation by altering the rumen microbiome and energy metabolism pathways [54]. Feng et al. found that PSE has a regulatory effect on gut flora and faecal metabolites in rats with NAFLD (nonalcoholic fatty liver disease) [22]. And in our research, we put PS and PSE together and made a comparison between these two supplementations and found that both PS and PSE exhibit similar probiotic effects, contributing to improvements in gut microbiota, growth promotion, and immune response. Despite some nuanced differences in their respective impacts, these findings establish a solid foundation for further optimizing the utilization of PS and PSE in the future.

Only one previous study has systematically cross-compared sterols and sterol esters in adults, where the depletion of esterified sterols and sterol mixtures resulted in reduced plasma TC concentrations, which were largely indistinguishable between the two groups [55]. The present study’s results indicate that PS are more effective than PSE in increasing TC and TG concentrations. Conversely, PSE demonstrate higher efficacy than PS in reducing the inflammatory chemokines CXCL1 and CXCL2 and in elevating SOD activity values. However, further studies on the effects and mechanisms of both compounds are needed.

## 5 Conclusions

In summary, our study found that diets enriched with PS and PSE enhance both growth and immune performance in mice, possibly by elevating Akkermansia levels in the intestine. However, additional research is needed to validate this hypothesis.

## Supporting information

S1 File (XLS)

S2 File (XLS)

S3 File (CSV)

S4 File (CSV)

S5 File (CSV)

S6 File (CSV)

S7 File (CSV)
